# Assessment of Genetic Diversity and Symbiotic Efficiency of Selected Rhizobia Strains Nodulating Lentil (*Lens culinaris* Medik.)

**DOI:** 10.3390/plants10010015

**Published:** 2020-12-24

**Authors:** Badreddine Sijilmassi, Abdelkarim Filali-Maltouf, Hassan Boulahyaoui, Aymane Kricha, Kenza Boubekri, Sripada Udupa, Shiv Kumar, Ahmed Amri

**Affiliations:** 1 Rhizobium Laboratory, Genetic Resources Section, International Center for Agricultural Research in the Dry Area (ICARDA), Agdal, Rabat 10080, Morocco; S.udupa@cgiar.org (S.U.); SK.Agrawal@cgiar.org (S.K.); A.amri@cgiar.org (A.A.); 2 Microbiology and Molecular Biology Laboratory, Faculty of Sciences, Mohammed V University, Rabat 1014, Morocco; filalimaltouf@gmail.com; 3 Center of Genomics of Human Pathologies (GENOPATH), Faculty of Medicine & Pharmacy, Mohammed V University, Rabat 10000, Morocco; hassan.boulahyaoui@um5s.net.ma; 4 Department of Biochemistry and Molecular Biology, Huazhong University of Sciences and Technology, Wuhan 430074, China; aymanekricha@hust.edu.cn; 5 AgroBio Sciences Program, Mohammed VI Polytechnic University (UM6P), Benguerir 43150, Morocco; boubekrikenza@yahoo.fr

**Keywords:** *Rhizobium*, rhizobia, lentil, genetic diversity, symbiotic efficiency, phylogenetic analysis, multi-locus sequence analysis (MLSA)

## Abstract

A total of 14 *Rhizobium* strains were isolated from lentil accessions grown at the ICARDA experimental research station at Marchouch in Morocco and used for molecular characterization and symbiotic efficiency assessment. Individual phylogenetic analysis using the 16S rRNA gene, house-keeping genes *rpoB*, *recA*, and *gyrB*, and symbiotic genes *nodD* and *nodA* along with Multilocus Sequence Analysis (MLSA) of the concatenated genes (*16S rRNA*-*rpoB*-*recA*-*gyrB*) was carried out for the identification and clustering of the isolates. The symbiotic efficiency of the strains was assessed on three Moroccan lentil cultivars (Bakria, Chakkouf, and Zaria) based on the number of nodules, plant height, plant dry weight, and total nitrogen content in leaves. The results showed that the individual phylogenetic analysis clustered all the strains into *Rhizobium laguerreae* and *Rhizobium leguminosarum* with sequence similarity ranging from 94 to 100%, except one strain which clustered with *Mesorhizobium huakuii* with sequence similarity of 100%. The MLSA of the concatenated genes and the related percentages of similarity clustered these strains into two groups of *Rhizobium* species, with one strain as a new genospecies when applying the threshold of 96%. For symbiotic efficiency, the Bakria variety showed the best association with 10 strains compared to its non-inoculated control (*p*-value ≤ 0.05), followed by Chakkouf and Zaria. The present study concluded that the genetic diversity and the symbiotic efficiency of *Rhizobium* strains appeared to be mainly under the control of the lentil genotypes.

## 1. Introduction

Lentil (*Lens culinaris* Medik.) is an important cool season food legume crop grown extensively in South Asia, West Asia, North Africa, North America, and Australia [1]. Lentil grains are used for human consumption and the dry straw as animal fodder. Lentil grains are rich in proteins, micronutrients, and prebiotics and play an important role in augmenting food and nutritional security. Lentil is grown in rotation with cereals to diversify rainfed cereal-based monocropping. Being a legume, the lentil has the ability to fix atmospheric nitrogen in its root nodules by symbiotic association with *Rhizobium*, thereby, it helps in improving soil fertility and health [2]. Nitrogen (N) is considered one of the most important nutrients for plant growth [3] and its deficiency in the soil affects drastically crop yield [4]. N is usually supplied through the application of mineral fertilizers. However, in addition to increased cost, there are close relationships between the application of nitrogen fertilizers and environmental problems such as eutrophication, the greenhouse effect, and acid rain [5]. Consuming contaminated groundwater or crops with a high concentration of nitrate has negative effects on human health [6]. Hence, biological nitrogen fixation is increasingly gaining importance in crop production [7].

Biological nitrogen fixation (BNF) is a phenomenon with which the nitrogen of the atmosphere is transformed into ammonia through an enzymatic reaction of soil bacteria [8]. More than 60% of soil nitrogen is provided through BNF [9]. The most important BNF activity in terms of the amount of nitrogen fixed is shown in the associations between rhizobia species and legumes [10]. Previous reports estimated that over 70 million tonnes of nitrogen are produced from the symbiotic nitrogen fixation (SNF) [11]. Therefore, SNF remains the appropriate solution to overcome low soil nitrogen fertility and to reduce the increasing demand for chemical nitrogen fertilizers [12,13,14]. SNF contributes to sustainable agricultural development by benefiting the succeeding crops within the rotations. Like other legumes, lentil forms a symbiotic association with specific rhizobia providing the plant with part or most of its nitrogen needs.

The success of the symbiotic association relies on the two partners. The SNF efficiency differs according to the nodulating host plant and the rhizobia triggering the nodulation regulated by specific biochemical communication between them [15]. There is a huge diversity of rhizobia covering more than 98 species belonging to 14 genera within the alpha-proteobacteria and beta-proteobacteria groups [16,17]. It is reported by many studies that *Rhizobium leguminosarum* is the main symbiotic partner of the *Viceae* group including lentils [18,19,20]. Recent studies in Bangladesh and Ethiopia reported that the *Rhizobium* species nodulating lentils in different geographic areas may belong to different species [21,22,23]. This revealed a large diversity of the lentil symbionts and could explain the low effect of *Rhizobium* strains used randomly as inoculum [24]. However, very little work has been done towards the identification of *Rhizobium* species nodulating lentils in Morocco and their cultivar specificity.

With the advent of genome sequencing, PCR-based methods with DNA sequencing have been widely used for the genetic diversity analysis of bacteria, especially for some groups which are difficult to be distinguished by conventional methods. The 16S rRNA gene is one of the most powerful gene markers used for the genotypic characterization of the bacterial population because of the universality of this gene in all bacterial genomes and also by its sequence size (1500 bp) made of conserved, variable, and hypervariable regions [25,26]. However, this marker showed its limitation in separating close rhizobial species of the genus *Rhizobium* [27,28]. A new genetic identification approach has been suggested based on the analysis of the multi-locus sequence (MLSA) of house-keeping genes such as *recA*, *gyrB*, and *rpoB* [29]. MLSA can detect genetic diversity within and between species since the difference is assigned at the nucleotide level [29].

The present study used the MLSA technique to assess the genetic diversity of the rhizobial strains isolated from lentil germplasm and assess their symbiotic efficiency on three varieties of lentil.

## 2. Results

### 2.1. Isolation and Symbiotic Efficiency of the Isolates

A total of 68 rhizobia strains were isolated from the nodules of 10 lentil accessions grown as a part of the lentil regeneration experiment conducted at the ICARDA experimental research station, Marchouch, Morocco (Table 1). Among these, 14 representative isolates were selected using the PCR-RFLP analysis of the genes *16S rRNA* and *nodD* to assess their symbiotic efficiency on three Moroccan varieties of lentil (Bakria, Chakkouf, and Zaria) (Table 1).

**Figure 1:**
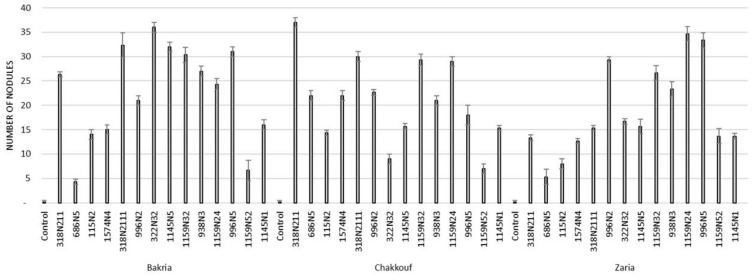
Number of nodules formed in three lentil varieties (Bakria, Chakkouf, Zaria) in association with 14 selected rhizobia isolates. The values are the mean of three replicates. (Ⅰ) standard deviation.

**Figure 2:**
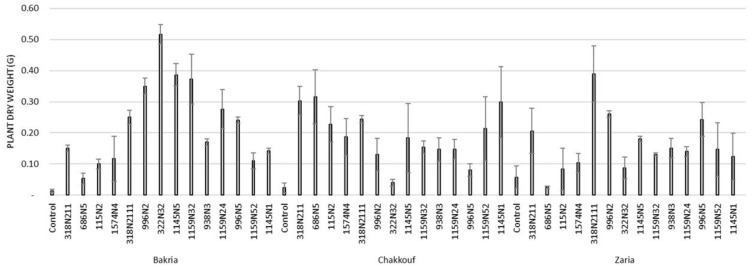
Plant dry weight (G) of three lentil varieties (Bakria, Chakkouf, Zaria) in association with 14 selected rhizobia isolates. The values are the mean of three replicates. (Ⅰ) standard deviation.

**Figure 3:**
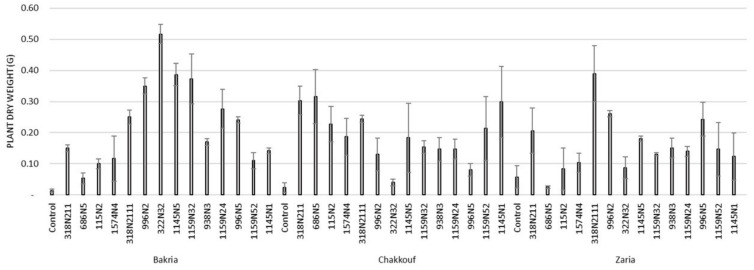
Plant height (cm) of three lentil varieties (Bakria, Chakkouf, Zaria) in association with 14 selected rhizobia isolates. The values are the mean of three replicates (Ⅰ) standard deviation.

**Figure 4:**
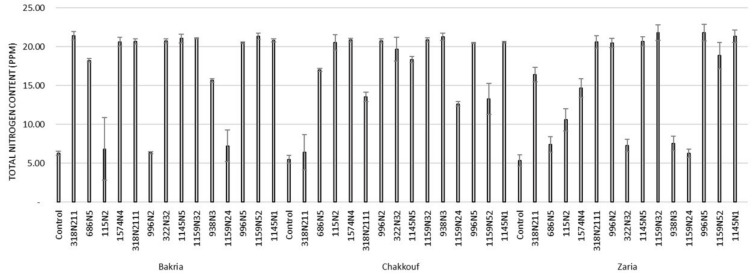
Total nitrogen content (PPM) in leaves of three lentil varieties (Bakria, Chakkouf, Zaria) in association with 14 selected rhizobia isolates. The values are the mean of three replicates (Ⅰ) standard deviation.

These 14 isolates were able to form a significant number of nodules on lentil varieties ranging between 4 and 37 nodules per plant (Figure 1 and Table S1). The equality of means test was found significantly different using both Welch and Brown–Forsythes tests with *p*-value ≤ 0.05. A total of 10 isolates had significant symbiotic efficiency on the Bakria variety based on the plant dry weight, plant height, and total nitrogen content in leaves (*p*-value ≤ 0.05), compared to the uninoculated control (Figure 2, Figure 3 and Figure 4). For the Chakkouf and Zaria varieties, only three isolates showed significant symbiotic efficiency based on the same parameters of growth. Partial and Bivariate correlations were positively significant between the parameters of growth for the isolate-Bakria associations, while no significant correlations were observed between total nitrogen content in the leaves and the other parameters of growth (number of nodules, plant dry weight, and plant height) for the isolate-Chakkouf and isolate-Zaria combinations (Tables S2–S4).

### 2.2. Gene Amplification and Sequencing

The degenerate primers recAF/recAR and rpoB83F/rpoB1061R generated several bands ranging between 500–3000 bp for the *recA* gene, and 500–1500 bp for the *rpoB* gene. Bands were cut and extracted from the gel using the extraction kit (PureLink, Quick Gel Extraction Kit; Invitrogen) following the instruction of the manufacturer. The targeted extracted bands were 500 bp and 990 bp for *recA* [30] and *rpoB* [31] genes, respectively. Single bands were generated from the amplification of the 16S rRNA (1500 bp) as well as from the symbiotic *nodD* (900 bp), and *nodA* (660 bp) genes [14,32,33,34].

The regions of the obtained fragments of the sequences after assembling and editing were 893–1037 bp for the 16S rRNA gene, 621–692 bp for the *gyrB* gene, 415–453 bp for the *recA* gene, 491–934 bp for the *ropB* gene, 600–663 bp for the *nodA* gene, and 784–872 bp for the *nodD* gene.

### 2.3. Individual Phylogenetic Analysis of the 16S rRNA Gene

**Figure 5:**
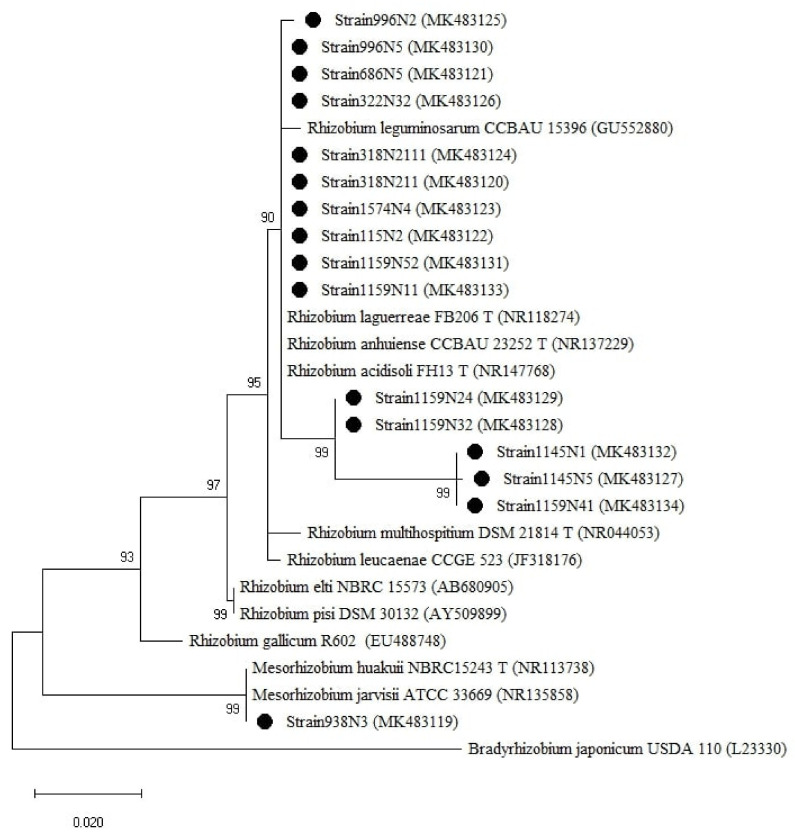
Phylogenetic tree built based on the individual analysis of the partial 16S rRNA gene sequences (807 bp) of the selected isolates and the closest sequences in terms of Max Scores on the NCBI website. The tree was generated using MEGAX with the Kimura 2-parameter (K2) distance model, and Maximum Likelihood (ML) method with the 1000 bootstrap analysis and bootstrap value (B.V ≥ 50%). The isolates were out grouped by *Bradyrhizobium japonicum* USDA110. Bars: 2% nucleotide substitutions. ● represents the sequences of the selected isolates.

The isolates were clustered into two groups based on the 16S rRNA genes (Figure 5), one group belonged to the genus *Rhizobium* with sequence similarity ranging between 97–100% with *R. anhuiense* CCBAU23252T, *R. laguerreae* FB206T, *R. leguminosarum* CCBAU15396, *R. acidisoli* FH13T. The strain 1159N11 presented 97% similarity with *R. leucaenae* CCGE 523. The second group is related to the genus *Mesorhizobium* where the isolate 938N3 showed 100% similarity with *M. huakuii* ATTCC 33669T and *M. jarvisii* ATCC 33669T (Table A1).

### 2.4. Individual Phylogenetic Analysis Based on House-Keeping and Symbiotic Genes

**Figure A1:**
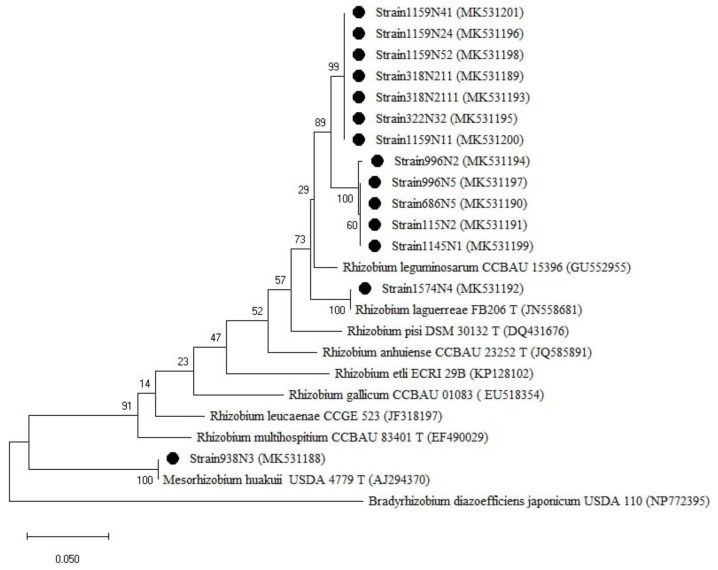
Phylogenetic tree built based on the individual analysis of the partial *recA* gene sequences (413 bp) of the selected isolates and the closest sequences in terms of Max Scores on the NCBI website. The tree was generated using MEGAX with the Kimura 3-parameter (T92) distance model and Maximum Likelihood (ML) method with the 1000 bootstrap analysis and bootstrap value (B.V ≥ 50%). The isolates were out grouped by *Bradyrhizobium japonicum* USDA 110. Bars: 5% nucleotide substitutions. ● represents the sequences of the selected isolates.

For the *recA* gene tree (Figure A1), the isolates were also divided into two groups, one related to the genus *Rhizobium* with a percentage of similarity ranging from 96 to 100% with *R. laguerreae* FB206T and 95 to 100% with *R. leguminosarum* CCBAU15396. Strain 1574N4 was the closest isolate to *R. laguerreae* FB206T with 100% similarity. The second group was related to the genus *Mesorhizobium* where strain 938N3 showed 100% similarity with *M. huakuii* USDA4779T (Table A1).

**Figure A2:**
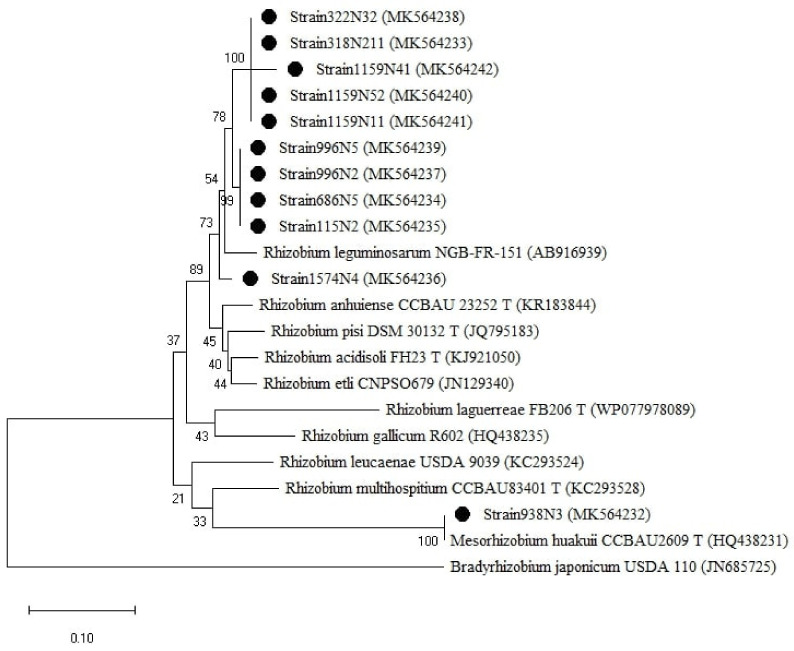
Phylogenetic tree built based on the individual analysis of the partial *gyrB* gene sequences (571 bp) the selected isolates and the closest sequences in terms of Max Scores on the NCBI website. The tree was generated using MEGAX with the Kimura 3-parameter (T92) distance model and Maximum Likelihood (ML) method with the 1000 bootstrap analysis and bootstrap value (B.V ≥ 50%). The isolates were out grouped by *Bradyrhizobium japonicum* USDA 110. Bars: 50% nucleotide substitutions. ● represents the sequences of the selected isolates.

For the *gyrB* gene tree (Figure A2), the isolates were divided into two groups, one group related to *Rhizobium* in which the isolates were divided into three sub-groups with a percentage of sequence similarity ranging between 94 and 97% with *R. leguminosarum* NGB-FR-141 and the second group related to the genus *Mesorhizobium* where strain 938N3 showed 100% similarity with *M. huakuii* CCBAU2609 (Table A1).

**Figure 6:**
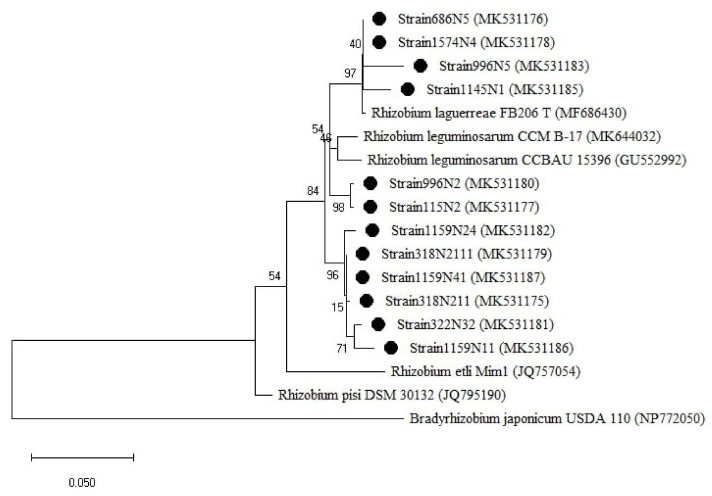
Phylogenetic tree built based on the individual analysis of the partial *rpoB* gene sequences (477 bp) of the selected isolates and the closest sequences in terms of Max Scores on the NCBI website. The tree was generated using MEGAX with the Kimura 3-parameter (T92) distance model and Maximum Likelihood (ML) method with the 1000 bootstrap analysis and bootstrap value (B.V ≥ 50%). The isolates were out grouped by *Bradyrhizobium japonicum* USDA 110. Bars: 10% nucleotide substitutions. ● represents the sequences of the selected isolates.

**Figure 7:**
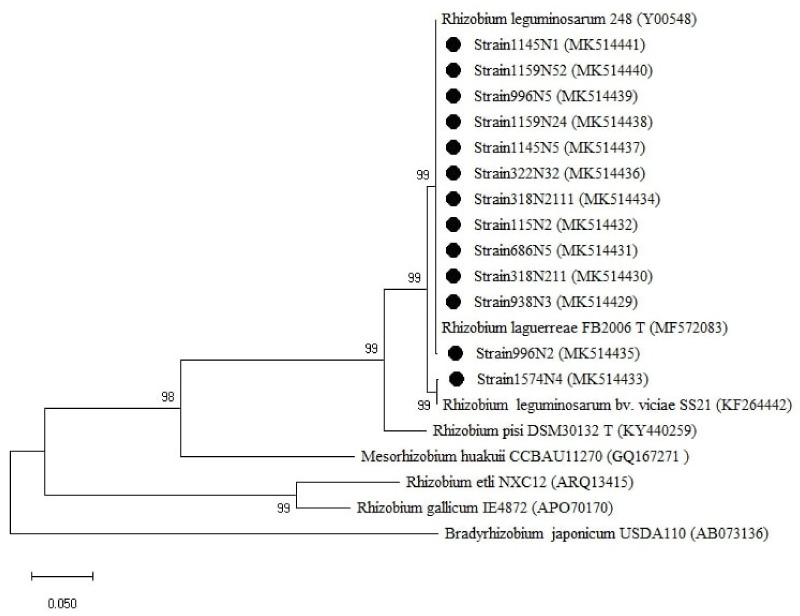
Phylogenetic tree built based on the individual analysis of the partial *nodD* gene sequences (758 bp) of the selected isolates and the closest sequences in terms of Max Scores on the NCBI website. The tree was generated using MEGAX with the Kimura 2-parameter (K2) distance model and Maximum Likelihood (ML) method with the 1000 bootstrap analysis and bootstrap value (B.V ≥ 50%). The isolates were out grouped by *Bradyrhizobium japonicum* USDA110. Bars: 5% nucleotide substitutions. ● represents the sequences of the selected isolates.

**Figure A3:**
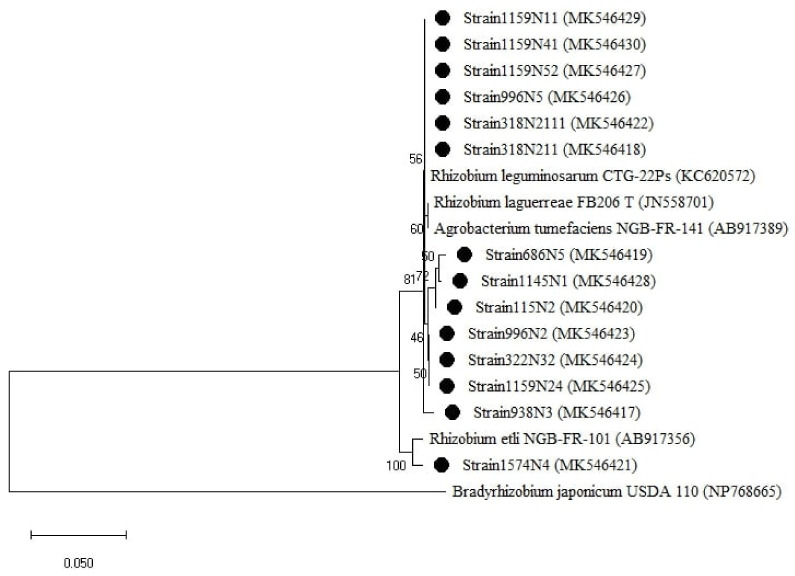
Phylogenetic tree built based on the individual analysis of the partial *nodA* gene sequences (600 bp) of the selected isolates and the closest sequences in terms of Max Scores on the NCBI website. The tree was generated using MEGAX with the Kimura 3-parameter (T92) distance model and Maximum Likelihood (ML) method with the 1000 bootstrap analysis and bootstrap value (B.V ≥ 50%). The isolates were out grouped by *Bradyrhizobium japonicum* USDA 110. (EU420074). Bars: 5% nucleotide substitutions. ● represents the sequences of the selected isolates in the study.

For the *rpoB* gene tree (Figure 6), the isolates were all clustered with the *Rhizobium* group, which was divided into two sub-groups. One sub-group related to *R. laguerreae* FB206T with a percentage of similarity ranging from 96 to 100% and the second sub-group was related to *R. leguminosarum* CCBAU15396 with a percentage of similarity ranging from 96 to 97% (Table A1). Regarding the symbiotic genes, the isolates were all grouped within the genus *Rhizobium* (Figure 7 and Figure A3) with a percentage of similarities ranging from 96 to100% with *R. laguerreae* and 97 to 100% with *R. leguminosarum* in *nodA* and *nodD* genes, respectively (Table A1).

For *nodD* (Figure 7), the tree showed two groups related to the genus *Rhizobium* with the first cluster related to *R. leguminosarum bv. viciae* SS21 represented by one strain (1574N4) and the second cluster related to *R. leguminosarum* 248 and *R. laguerreae* FB206T.

For *nodA* (Figure A3), the isolates were grouped into three clusters, with cluster one related to *R. leguminosarum* CTG-22Ps, cluster two related to *R. laguerreae* FB206T, and cluster three represented with the strain 1574N4 related to *R. elti* NGB-FR-141. Unexpectedly, *R. laguerreae* FB206T and strain 1574N4 were clustered with *Agrobacterium tumefaciens* NGB-FR-141 and *R. elti* NGB-FR-101, respectively, despite their host-specificity group divergence.

### 2.5. Multi-Locus Sequence Analysis (MLSA) of the Concatenated Genes (16S rRNA-rpoB-recA-gyrB)

**Figure 8:**
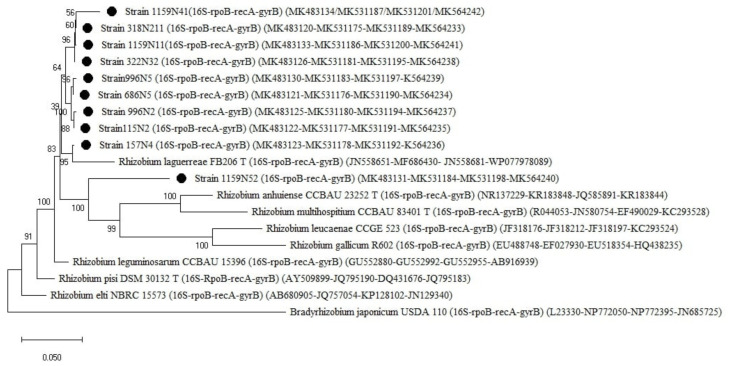
Phylogenetic tree built based on the concatenated gene sequences *(16S rRNA-rpoB-recA-gyrB)* (2194 bp) of the selected isolates and the reference Rhizobium species retrieved from the NCBI website database. The tree was generated using MEGAX with the general time-reversible distance model and Maximum Likelihood (ML) method with the 1000 bootstrap analysis and bootstrap value (B.V ≥ 50%). The isolates were out grouped by *Bradyrhizobium japonicum* USDA 110. Bars: 5% nucleotide substitutions. ● represents the isolates sequences.

The MLSA of the concatenated genes (*16S rRNA-rpoB-recA-gyrB*) showed three groups, all related to *Rhizobium* (Figure 8). The first group, represented by the strain 1574N4, was closely related to *R. laguerreae* FB206T with 96% similarity. The second group was related to *R. leguminosarum* NGB-FR-151 and can be further subdivided into two sub-groups with a percentage of similarity ranging between 94 and 97%. The third group was represented by the strain 1159N52. The strains 159N52 and 1159N41 showed the lowest similarity of 95 and 96%, respectively, to *R. leguminosarum* NGB-FR-151 (Table A1).

### 2.6. Topological Congruence of the Phylogenetic Trees

In general, the congruency index Icong test [35] showed no topological congruence between the trees (individual and concatenated trees) except for the concatenated (*16S rRNA*-*rpoB*-*recA*-*gyrB*) and *gyrB* phylogenetic trees with Icong = 1.53 (*p*-value = 0.001) (Table 2). The closest topological congruence between individual trees was shown between the *recA* and *gyrB* phylogenetic trees with Icong = 1.25 and *p*-value = 0.006 (Table 2).

### 2.7. Loci Priorities

The lengths of the loci after alignment were 807 bp, 477 bp, 413 bp, 571 bp, 600 bp, 758 bp, and 2194 bp for *16S rRNA*, *rpoB*, *recA*, *gyrB*, *nodA*, *nodD* and concatenated genes (*16S rRNA-rpoB-recA-gyrB*), respectively (Table 3). Among the genes studied, the *rpoB* gene was the most variable within the population of the isolates based on the percentage of variable sites, Distance Range, Pairwise Distance Mean, and Transition/Transversion index, followed by the genes *gyrB* and *recA.* The symbiotic genes (*nodA* and *nodD*) showed higher conservation compared to the protein-coding housekeeping genes (*rpoB*, *gyrB*, and *recA*) with *nodA* and *recA* being the most conserved among the studied symbiotic and house-keeping genes (Table 3).

## 3. Discussion

### 3.1. Characterization and Phylogenetic Relationships of the Isolated Rhizobial Strains Based on the 16S rRNA, House-Keeping and Symbiotic Genes

Several studies reported that *R. leguminosarum*, *R. elti*, and *R. laguerreae* are the predominant species of nodulating lentil, nonetheless, other *Rhizobium* species could also be found [21,22,23,28,36]. However, these studies focused only on the strains isolated from a given location or a region without considering the genotypic diversity of the host plant. In this study, the molecular characterization of the isolates collected from 10 different lentil germplasm accessions showed that *R. laguerreae, R. leguminosarum* and *M. huakuii* species were the symbionts nodulating lentils, suggesting that sampling of rhizobia from different genotypes of lentil is needed to capture efficiently the different species of *Rhizobium* nodulating lentil. A similar result was obtained for *Bradyhizobium*-soybean associations by Lindström et al. and Ribeiro, et al. [37,38].

To have a comprehensive insight into the isolated rhizobial population diversity, a phylogenetic analysis was carried out using different gene markers on 14 representative strains selected from 10 different lentil accessions. The *16S rRNA* gene phylogenetic analysis clustered the analyzed strains into one *Rhizobium* group which included *R. anhuiense*, *R. laguerreae*, *R. leguminosarum*, and *R. acidisoli* with sequence similarity ≥97% (Figure 5 and Table A1). Many studies have shown that the gene *16S rRNA* is not able to separate closely related species such as *R. leguminosarum*, *R. multihospitium*, *R. minosarum*, and *R. freirei* [23,27,39]. The use of the phylogenetic analysis of house-keeping and symbiotic genes has brought greater resolution as also reported by Tindall et al. [40]. Some of the strains were positioned differently in the phylogenetic trees of different genes. For instance, strain 1574N4 was positioned close to *R. laguerreae* for *rpoB* and *recA*, close to *R. leguminosarum* for *gyrB* and *nodD*, and close to *R. elti* for *nodA*. This might be explained by the different evolutionary histories of the genes [41] due to environmental selective pressure [42]. The genetic diversity analysis of the sequence data showed different variability and diversity among the genes within a species. The symbiotic genes were more conserved compared to the house-keeping genes within the isolated population. This might be due to the high rate of HGT (horizontal genetic transfer) among the populations that coexist at the same place, considering that *nodD* and *nodA* are genes localized in the transmissible large plasmid (Sym plasmid) [43]. The *rpoB* gene showed the highest variability rate, expressed by Transition/Transversion index, genetic distance range and pairwise distance mean index, compared to *recA* and *gyrB* genes within the studied population. This finding is supported by the nature of each gene with *rpoB* containing hyper-variable zones allowing for rapid identification of bacteria at a sub-species level [44] whereas *recA* and *gyrB* genes, widely used for interspecies rhizobia classification [45,46] are considered very conserved genes and useful in discriminating between species [47]. Hence, the *rpoB* gene remains the best marker to be used for studying the relationships between closely related strains [29].

Interestingly, the phylogenetic trees of the *16S rRNA*, *recA*, and *gyrB* clustered the strain 938N3 into *Mesorhizobium* with 100% similarity with *M. huakuii* and *M. jarvis* for the 16S rRNA gene and 100% similarity with *M. huakuii* for *recA* and *gyrB* genes. A similar finding was reported by Dhaoui et al. [47] where the strain LB4 was isolated from lentil root nodules growing in various geographical regions of Tunisia. However, the phylogenetic analysis of the *nodD* gene sequences of 938N3 is similar to those of the symbiovar viciae strains within the species *R. leguminosarum* and *R. laguerreae*. This extensive incongruence between the phylogenetic analysis of the housekeeping genes and symbiotic genes was previously noted between *Rhizobium* and *Mesorhizobium* [48]. Lemaire et al. [48] found a *Rhizobium* strain carrying *nodA* and *nifH* symbiosis genes typical of *Mesorhizobium* strains, suggesting that a putative transfer might have occurred.

Although the clustering of the isolated strains was similar for some genes, for instance, *rpoB* and *recA* genes, the congruency index Icong test results showed no topological congruence between the individual trees of *rpoB*, *recA*, and *gyrB* genes. Similar results showing topological discordance between genes were reported in other studies [49,50]. Ludwig and Klenk [41] explained that the topological congruence of phylogenetic trees of different genes should not be expected, because one missing phylogenetic information or changes at the alignment level could significantly impact the outcome. However, the only significant congruence found was between the concatenated phylogenetic tree *(16S rRNA-rpoB-recA-gyrB)* and *gyrB* phylogenetic tree with congruency index Icong = 1.53 (*p*-value = 0.001). This might be due to the highly conserved regions of the *gyrB* gene and the ability of the MLSA to alleviate the effect of HGT between genes. This finding showed that the *gyrB* gene could be as robust as MLSA in clustering strains from the closest species.

For a higher resolution and to alleviate the HGT distortion (Rong and Huang, 2014) [29], MLSA was carried out based on the *16S rRNA* and the three (*rpoB*, *recA*, *gyrB*) housekeeping genes. The phylogenetic analysis of the concatenated genes (*16S rRNA-rpoB-recA-gyrB*) clustered 14 strains into three groups related to *Rhizobium* with two groups closely related to *R. laguerreae* and *R. leguminosarum* and a third group represented with a unique isolate (1159N52). The strain 1159N52 and strain 1159N41 showed the lowest similarity among the strains with 95 and 96% similarities to *R. leguminosarum*, respectively. Many studies considered 97% as the threshold for defining a new genospecies [46,51]. However, the difference in the utilized concatenated genes in the MLSA approach may impact the reliability of the threshold considering the independent evolutions of the genes. For this, we proposed to evaluate the threshold defining new genospecies by considering the percentage of similarity of the used reference strains based on the concatenated used genes *(16S rRNA-rpoB-recA-gyrB)* (Table 4). Based on the results, the strain 1159N52 could be considered as a potential new genospecies applying the threshold of 96% of the closest species (*R. leguminosarum*). Nevertheless, further investigations are needed to confirm these findings by studying additional housekeeping genes and whole genome sequencing [52,53].

### 3.2. Assessment of Symbiotic Efficiency

The selected strains were assessed for their symbiotic efficiency with three Moroccan lentil varieties, namely Bakria, Chakkouf, and Zaria. Bakria, registered in 1984 in Morocco, is the predominant variety used by the Moroccan farmers in several regions in the country. This choice comes from the fact that this variety carries many desirable agronomic traits such as high yield, large grain size, early maturity, and tolerance to rust and drought stress [54]. Zaria and Chakkouf varieties, registered in 2003 and 2009 respectively in Morocco, were proposed because of their mechanical harvesting ability [55] and their resistance to Ascochyta blight disease caused by *Ascochyta fabae.* f. sp. *lens* [56].

In this study, the Bakria variety showed the best associations with most of the isolated strains of rhizobia in terms of the number of nodules, plant height, plant dry weight, and total nitrogen content in leaves, followed by Chakkouf and Zaria varieties. This result might be explained by the adaptability and the coevolution of the lentil genotype and rhizobia population [57]. The Bakria variety, because of its larger area of cultivation, seems to be more adapted to the indigenous rhizobial population than Chakkouf and Zaria varieties grown in limited acreage. Ferguson et al. [58] reported that the legume host controls the whole symbiotic process, starting from the rhizobial invasion, nodulation, and even nitrogen regulation within the nodules. Thus, selection for SNF efficiency in the lentil breeding program could increase biological nitrogen fixation and decrease the amount of chemical fertilizer application in lentil production.

Partial and bivariate correlations were significantly positive between the combinations of four growth parameters for the isolate-Bakria associations (*p*-value ≤ 0.05). However, no significant correlations were found between the total nitrogen content in leaves and other parameters of growth (number of nodules, plant dry weight, and plant height) for the isolate-Chakkouf associations and isolate-Zaria associations. SNF and nodulation are two independent activities regulated by different sets of genes. SNF is mainly controlled by the *nifH* gene encoding the nitrogenase enzyme [59] whereas nodulation is the result of nod genes expression regulated by the *nodD* gene [60] and consequently a high number of nodules does not necessarily imply a high total nitrogen content

## 4. Materials and Methods

### 4.1. Rhizobium Sampling Site and Plant Materials

In total, 10 lentil accessions were grown as a part of the lentil regeneration experiment conducted at the ICARDA experimental research station, Marchouch, Morocco (Longitude: 33.561319; Latitude: −6.691883; Altitude: 428 m) were used for nodule collection (Table 1). The soil of the field experiment (0–20 cm) was of clay texture composed of clay (64.5%), fine loam (10.8%), sandy loam (12%), and sand (4.5%). The other soil characteristics were pH H_2_O: 6.5; pH KCl: 5.5; organic matter: 1.6%; K_2_O: 210.9 mg·kg^−1^; P_2_0_5_: 45.9 mg·kg^−1^ and EC: 0.30 mS·cm^−1^ and Total N: 0.085%.

### 4.2. Nodule Collection and Isolation of Bacteria

The collection of nodules was made in April/May 2016 during the flowering stage. Root nodules were collected from 10 different accessions of lentils following the procedure of Prévost and Antoun [61]. The root part of the plants was pulled out of the soil, roots were thoroughly washed with tap water, and nodules were collected. The collected nodules were washed under tap water and surface sterilized by soaking them in 95% (*v*/*v*) ethanol for 10 s, followed by 5% (*w*/*v*) sodium hypochlorite for 1 min, and finally rinsed five times with sterilized distilled water. The sterilized nodules were crushed onto the center of an empty sterile petri dish. A drop of sterilized distilled water was added to each crushed nodule and the suspension was streaked onto Yeast Extract-Mannitol Agar (YMA) [62]. Purification of isolates was done through successive sub-culturing using the YMA medium. Each purified isolate was maintained in YMB with 25% (*w*/*v*) glycerol at −80 °C for long term storage.

### 4.3. Plant Inoculation and Symbiotic Efficiency Assessment

Three varieties of lentil released in Morocco, namely Bakria (ILL4605), Chakkouf (ILL6001), and Zaria (ILL6021) were used as hosts for the nodulation and symbiotic efficiency tests. The seeds were surface sterilized following the procedure described by Somasegaran and Hoben [63]. The seeds were washed several times by tap water, then soaked in 70% (*v*/*v*) ethanol for 5 min and in 5% sodium hypochlorite for 2 min with agitation followed by soaking them in sterilized distilled water for 30 min.

Bacterial suspensions were prepared for plant inoculation by growing the isolates in YM Broth [63] at 28 °C and 150 rpm for 48 h. The density of the bacterial cells were estimated using UV/VIS spectrophotometer (T80, PG-Instruments) and diluted to ≈ 1.0 at OD 600 nm, which is equal to 10^8^ CFU/mL. [64]. The seed inoculation was performed following the procedure of Howieson and Dilworth [65]. Seeds were inoculated with 1 mL of the bacterial suspension after being pre-germinated for two days under dark at 22 °C. The soil used in this experiment was brought from Marchouch station. The soil was sterilized by autoclaving at 30 min at 130 °C three times with one-day intervals between autoclave cycles. Polyethylene pots (25 cm diameter × 25 cm height) were sterilized by washing them in 20% (*w/v*) sodium hypochlorite, filled with 1 kg of the sterilized soil. In each pot, one plant of each of the three lentil varieties was planted and inoculated with the same strain. An uninoculated pot planted with three varieties was used as a negative check. Each strain and negative control were replicated three times following a completely randomized block design. Pots were placed under control conditions in the greenhouse with 26 °C day/22 °C night temperature and 16 h day/8 h night photoperiod and were irrigated using nitrogen-free sterilized nutrient solution [66]. At the flowering stage, plant dry weight (g), number of nodules, plant height (cm) were assessed, and the total nitrogen content in leaves (mg/mL) was determined using the Kjeldahl digestion method [67] (Table S1).

### 4.4. Statistical Analysis

Descriptive statistics, along with ANOVA and correlations were done using SPSS 20 [68]. The test of homogeneity of variances and the robust tests of equality of means (Welch’s test and Brown-Forsythe’s test) were performed. The associations of variety-strain were compared using the Tukey HSD test. For each variety, across all strains, the correlations between the growth parameters measured were calculated using Pearson’s correlation coefficient.

### 4.5. DNA Extraction, PCR Amplification, and Gel Electrophoresis

A total of 68 isolates were grown in the R2A Medium [69] for two days at 28 °C to avoid exopolysaccharide production during bacteria growth. The DNA extraction was carried out following the method described by Kowalchuk et al. [70] with SDS/CTAB as a buffer of digestion and phenol/chloroform for extraction. The concentration and the purity of the DNA (Ratios A260/A280 and A260/A230) [71] were assessed using the Nanodrop spectrophotometer (Jenway, Genova nano).

In total, 14 isolates were selected to represent the 10 lentil genebank accessions (Table 1) and based on the genetic diversity revealed by PCR- RFLP analysis of the *16S rRNA* with *Msp*I, *Hae*III, and *Hin*fI restriction enzymes and the PCR-RFLP analysis of *nodD* with *Alu*I restriction enzyme was carried out. *Rhizobium sp.* CIAT988 [72] was taken as a reference species.

The small unit 16S rRNA and three house-keeping genes, *gyrB* (DNA gyrase), *rpoB* (beta-subunit RNA polymerase) and *recA* (DNA recombinase A), and two symbiotic genes, *nodD* (nod-genes Box expression regulator), and *nodA* (N-acyltransferase) were amplified by PCR using corresponding primers (Table 5). PCR amplifications were performed with the thermocycler ((MultiGene Optimax, Labnet). The PCR reactions were performed using Master Mix (MyTaq Mix, Bioline, London, UK) and 10–50 ng·uL^1^ of template DNA (Table 5). The PCR products were run on an agarose gel (0.8–1.2%) and molecular sizes of the amplified fragments were estimated using 1 kb HyperLadder (Bioline).

### 4.6. Sequencing, and Phylogenetic Analysis

PCR products were purified using the ExoSAP-IT purification system (Cleanup Reagent, Invitrogen, Carlsbad, CA, USA) according to the manufacturer’s protocol. Sequencing was carried out using Big dye Terminator cycle sequencing Kit V3.1 (Applied Biosystems, Waltham, Massachusetts, USA) in both forward and reverse directions with the same primers used for PCR. The sequence data were collected from an ABI 3730XL DNA capillary sequencer (Applied Biosystems) at NeoBiotech, France. The obtained nucleotide sequences were blasted using the nucleotide basic local alignment tool (BLASTN) queuing system 2.2.28 [75].

For phylogenetic analysis, nucleotide sequences of related strains for each studied gene were retrieved from GenBank and aligned using the ClustalW program in MEGA X ver. 10.1 software [76]. Once aligned, Maximum Likelihood (ML) trees were constructed using MEGA X software [77] with the suggested best model. The statistical significance of the nodes was assessed by bootstrap resampling analysis (1000 replicates) [76].

The assessment of the existence of topological congruence between the trees was conducted by using the online calculation of the congruency index Icong [35] (Table 2). The intergenic/intragenic sequence similarity calculation was carried out with the SIAS (Sequence Identity and Similarity) tool (http://imed.med.ucm.es/Tools/sias) using the BLOSUM62 alignment score matrix method [78] (Table 3). Locus properties were studied with MEGA X by the calculation of different statistical analysis parameters, including the Composition Distance Average test, Disparity Index Average test, Variable sites (%), Mean G + C Content (mol %), Transition/Transversion test, and Pairwise Distance Mean test (Table 3).

For the MLSA, a Maximum Likelihood tree was constructed using concatenated sequences of the selected genes (*16S rRNA*-*rpoB*-*recA*-*gyrB*). The isolate sequences were compared with those of nine reference strains: *Rhizobium acidisoli* strain FH13T, *Rhizobium laguerreae* strain FB206T, *Rhizobium leucaenae* strain CCGE523, *Rhizobium pisi* strain DSM30132T, *Rhizobium anhuiense* strain CCBAU23252T, *Rhizobium elti* strain NBRC15573, *Rhizobium gallicum* strain R602, *Rhizobium leguminosarum* strain NGB-FR-151, and *Rhizobium multihospitium* strain CCBAU83401T.

### 4.7. Accession Numbers

All the sequences used in this study were registered with the NCBI (National Center for Biotechnology Information) under the following accession numbers *16S rRNA* (MK483119 to MK483134); *recA* (MK531188 to MK531201); *gyrB* (MK564232 to MK564242); *rpoB* (MK531175 to MK531187); *nodD* (MK514429 to MK514441); *nodA* (MK546417 to MK546430).

## 5. Conclusions

Our results showed that lentil germplasm can nodulate with several rhizobia species but predominantly with *R. leguminosarum.* However, one of the selected strains was clustered with the genus *Mesorhizobium*, which could be explained by the fact that this strain is carrying the *nodD* gene sequences similar to those of the symbiovar *viciae* strains within the *R. leguminosarum* species. The symbiotic efficiency tests based on growth parameters of three lentil varieties showed that the efficiency performance of a given strain depended on the nodulating host genotype. Therefore, selection for the symbionts should consider the host plant genotype.

Further investigations are needed to study the effects of locations and the environmental conditions on the diversity and the symbiotic efficiency of the rhizobia species nodulating lentils using both physiological tests along with parameters of growths. Thus, planting the same lentil germplasm in different geographical locations under different environmental conditions may bring more understanding of the rhizobia-host genotype relationships and will allow selection of the best associations for better efficiency under different environmental stresses.

## Figures and Tables

**Table 1:** List of rhizobial isolates and their attributed lentil accessions. The selected strains are in **bold**.

Lentil Accession	Origin	Number of Isolates	Isolates Number (#)
IG 69452	NA ^1^	26	**1159N11**; 1159N112; 1159N12; 1159N12; 1159N13; 1159N14; 1159N15; 1159N21; 1159N22; 1159N23; **1159N24**; 1159N25; 1159N31; 1159N32; 1159N33; 1159N34; 1159N35; 1159N43; 1159N44; 1159N45; 1159N51; **1159N52**; 1159N53; 1159N54; **1159N541**; 1159N55
IG 73856	NA	5	1574N13; 1574N2; 1574N3; **1574N4**; 1574N52
IG 789	Egypt	6	318N1; 318N21; **318N211**; **318N2111**; 318N42; 318N42
IG 1737	Ethiopia	4	**996N2**; 512N13; 512N3; 512N4
IG 315	Ethiopia	5	115N1; **115N2**; 115N3; 115N4; 115N5
IG 69452	NA	5	**1145N1**; 1145N2; 1145N3; 1145N4; 1145N5
IG 795	Egypt	5	322N1; 322N2; **322N32**; 322N5; 322N51
IG 4351	Iran	5	938N1; 938N2; **938N3**; 938N4; 938N13
IG 2292	Iran	4	686N3; 686N4; 686N1; **686N5**
IG 4774	Romania	3	512N1; 996N4; **996N5**

^1.^ Not Available.

**Table 2:** Topological congruence between trees of the 16S rRNA, *gyrB*, *recA*, *rpoB*, *nodD*, and *nodA* genes sequence calculated using the congruency index Icong [35].

Genes	*nodD*		*16S rRNA-rpoB-recA-gyrB*		*16S rRNA*		*gyrB*		*rpoB*	
	Icong	*p*-Value	Icong	*p*-Value	Icong	*p*-Value	Icong	*p*-Value	Icong	*p*-Value
***nodA***	1.11	0.38			-	-	-	-	-	-
***16S rRNA***	-	-	0.88	7.45	-	-	-	-	-	-
***gyrB***	-	-	1.53	0.001	1.04	0.91	-	-	-	-
***rpoB***	-	-	1.09	0.48	1.15	0.22	0.88	7.45	-	-
***recA***	-	-	1.09	0.48	0.92	4.48	1.25	0.06	0.77	34.41

**Table 3:** Genetic diversity and the variability among the aligned locus (*16S rRNA*, *rpoB*, *recA*, *gyrB*, *nodA*, *nodD*) through statistical analysis of the sequences. AL: allele length (bp) after alignment; NVS: No. of variable sites; vs. (%): variable sites (%); G + C (%): Mean G + C Content (mol %); R: Transition/Transversion index; DR.: Distance Range and P. DM.: Pairwise Distance Mean calculated with Kimura 2-parameter (K2) distance model. The numbers in bold represent the high values among the table.

Locus	AL (bp)	NVS	VS (%)	G + C (%)	DR	PDM	R
*16S rRNA*	807	89	11.03	52.30	0–0.096	0.04	0.50
*rpoB*	477	332	69.60	59.11	0–2.040	0.32	2.75
*recA*	413	75	18.16	62.62	0–0.184	0.04	1.75
*gyrB*	571	152	26.62	59.77	0–0.310	0.07	0.99
*nodA*	600	24	4.00	57.99	0–0.034	0.01	0.91
*nodD*	754	20	2.65	55.12	0–0.035	0.01	1.34
*16S rRNA-rpoB-recA-gyrB*	2194	648	16.55	58.53	0–0.168	0.06	1.13

**Table 4:** Percentage of sequence similarity (%) between the Rhizobium species based on the concatenated genes (*16S rRNA-rpoB-recA-gyrB*) conducted by the SIAS (sequence Identity and similarity) tool by using the Blosum62 method.

	*R. acidisoli* FH23T	*R. laguerreae* FB206T	*R. elti* CNPSO679	*R. anhuiense* CCBAU23252T	*R. leucaenae* USDA9039	*R. leguminosarum* CCBAU15396	*R. gallicum* R602	*R. multihospitium* CCBAU83401T	*R. pisi* DSM 30132T
*R. acidisoli* FH23T	-								
*R. laguerreae* FB206T	79%	-							
*R. elti* CNPSO679	78%	88%	-						
*R.anhuiense* CCBAU23252T	80%	93%	89%	-					
*R. leucaenae* USDA9039	79%	89%	88%	88%	-				
*R. leguminosarum* CCBAU 15396	80%	96%	90%	94%	88%	-			
*R. gallicum* R602	79%	88%	88%	89%	92%	88%	-		
*R. multihospitium* CCBAU83401T	80%	89%	89%	89%	93%	89%	90%	-	
*R. pisi* DSM30132T	79%	96%	90%	94%	90%	98%	90%	89%	-

**Table 5:** List of primers and PCR programs used for PCR amplification and sequencing of *16S rRNA*, housekeeping and symbiotic genes of the isolates. H = Adenine, Cytosine or Thymine; N = Adenine, Cytosine, Guanine or Thymine; Y = Cytosine or Thymine; W = Adenine or Thymine; M = Adenine OR Cytosine; K = Guanine or Thymine; S: Cytosine, Guanine; R = Adenine or Guanine.

Targeted Genes	Primers	Sequences	PCR Conditions	Reference
***16S rRNA***	16S 27F 16S 1492R	5′-AGAGTTTGATCCTGGCTCAG-3′ 5′-ACGGTTACCTTGTTACGACTT-3′	5 min 95 °C, 30 × (45 s 95 °C, 45 s 50 °C, 2 min 72 °C), 10 min 72 °C	[73]
***recA***	recAF recAR	5′-ATCGAGCGGTCGTTCGGCAAGGG-3′ 5′-TTGCGCAGCGCCTGGCTCAT-3′	5 min 95 °C, 35 × (45 s 95 °C, 60 s 53 °C, 40 s 72 °C), 7 min 72 °C	[73]
***gyrB***	gyrB343F gyrB1043	5′-TTCGACCAGAAYTCCTAYAAGG-3′ 5′-AGCTTGTCCTTSGTCTGCG-3′	5 min 95 °C, 35 × (45 s 95 °C, 60 s 53 °C, 40 s 72 °C), 7 min 72 °C	[74]
***rpoB***	rpoB83F rpoB1061R	5′-CCTSATCGAGGTTCACAGAAGGC-3′ 5′-AGCGTGTTGCGGATATAGGCG-3′	5 min 95 °C, 30 × (45 s 95 °C, 45 s 46 °C, 2 min 72 °C), 10 min 72 °C	[74]
***nodA***	nodAF nodCAR	5′-TGCRGTGGAARNTRNNCTGGGAAA-3′ 5′-GNCCGTCRTCRAAWGTCARGTA-3′	5 min 95 °C, 30 × (45 s 94 °C, 1 min 50 °C, 2 min 72 °C), 7 min 72 °C	[74]
***nodD***	Y5 Y6	5′-ATGCGKTTYARRGGMCTN GAT CT-3′ 5′-CGCAWCCANATRTTYCCNGGRTC-3′	5 min 95 °C, 30 × (45 s 94 °C, 1 min 58 °C, 2 min 72 °C), 7 min 72 °C	[73]

## Supplementary Materials

The following are available online at https://www.mdpi.com/2223-7747/10/1/15/s1, Table S1: Symbiotic efficiency of the isolates on the three Moroccan lentil varieties (Bakria, Chakkouf, and Zaria).

## Appendix A

**Table A1:** Intergenic/intragenic similarities (%) of the isolates of *Rhizobium* species based on the *16S rRNA*, *gyrB*, *recA*, *rpoB*, *nodD*, and *nodA* genes conducted by the SIAS (sequence Identity and similarity) tool by using the Blosum62 method. Numbers in bold are the highest percentage of similarity within the gene; - represents the percentage of similarity <94% except for concatenated genes. NA represents the missing data (not obtained sequences).

Genes	Genus/Species	938N3	318N211	686N5	115N2	1574N4	318N2111	996N2	322N32	1159N24	1159N52	996N5	1145N1	1159N11	1159N41
***16S rRNA***	***R. anhuiense* CCBAU 23252T**	-	100%	100%	100%	100%	100%	100%	100%	99%	100%	100%	97%	98%	97%
	***R. laguerreae* FB206T**	-	100%	100%	100%	100%	100%	100%	100%	99%	100%	100%	97%	98%	97%
	***R. leguminosarum* CCBAU 15396**	-	100%	100%	100%	100%	100%	100%	100%	99%	100%	100%	97%	98%	97%
	***R. acidisoli* FH13T**	-	100%	100%	100%	100%	100%	100%	100%	99%	100%	100%	97%	98%	97%
	***R. leucaenae* CCGE523**	-	-	-	-	-	-	-	-	-	-	-	-	97%	-
	***M. huakuii* NBRC15243**	100%	-	94%	94%	94%	-	94%	-	-	-	-	-	-	-
***gyrB***	***M. huakuii* NBRC15243**	100%	-	-	-	-	-	-	-	-	-	-	-	-	-
	***R. leguminosarum* CCBAU15396**	-	96%	97%	97%	97%	NA	96%	96%	NA	96%	97%	NA	95%	94%
***recA***	***R. laguerreae* FB206T**	-	97%	96%	95%	100%	97%	95%	97%	-	97%	96%	96%	97%	97%
	***R. leguminosarum* CCBAU15396**	-	100%	100%	100%	96%	100%	100%	100%	-	100%	100%	100%	100%	100%
	***M. Huakuii* USDA4779 T**	100%	99%	97%	97%	97%	99%	97%	99%	-	99%	97%	97%	99%	99%
***rpoB***	***R. laguerreae* FB206T**	NA	97%	100%	97%	100%	97%	98%	97%	97%	97%	98%	98%	96%	97%
	***R. pisi* DSM30132T**	NA	95%	95%	95%	95%	95%	95%	95%	95%	95%	-	-	94%	95%
	***R. elti* Mim1**	NA	-	-	-	-	-	-	-	-	-	-	-	-	-
	***R. leguminosarum* CCBAU15396**	NA	97%	97%	97%	97%	97%	97%	97%	97%	97%	95%	96%	96%	97%
***nodD***	***R. leguminosarum bv. vicia* Strain SS21**	100%	99%	98%	97%	100%	98%	98%	98%	98%	98%	97%	98%	NA	NA
	***R. laguerreae* FB206T**	-	100%	100%	99%	97%	100%	99%	100%	100%	100%	99%	99%	NA	NA
***nodA***	***R. leguminosarum* CTG-22Ps**	-	100%	100%	100%	97%	100%	100%	100%	-	100%	100%	100%	100%	100%
	***R. laguerreae* R106**	-	98%	100%	100%	96%	98%	100%	98%	-	98%	100%	98%	98%	100%
***16S rRNA-rpoB-recA-gyrB***	***R. laguerreae* FB206T**	NA	96%	95%	94%	96%	NA	94%	94%	NA	93%	NA	94%	94%	93%
	***R. leguminosarum* CCBAU15396**	NA	97%	98%	97%	97%	NA	97%	97%	NA	95%	NA	97%	97%	96%
	***R. pisi* DSM30132T**	NA	96%	96%	96%	96%	NA	96%	96%	NA	94%	NA	95%	96%	94%
	***R. elti* CNPSO670**	NA	94%	94%	94%	94%	NA	94%	94%	NA	93%	NA	94%	94%	92%
