# Supplementary material for: Assessment of Genetic Diversity and Symbiotic Efficiency of Selected Rhizobia Strains Nodulating Lentil (Lens culinaris Medik.)
Source: Plants (Basel). 2020 Dec 24;10(1):15. doi: 10.3390/plants10010015 (PMC7823456; doi:10.3390/plants10010015)
Supplement: Supplementary file 1 [file plants-10-00015-s001.pdf]

Supplementary Materials:

Table S1. Symbiotic efficiency of the isolates on the three Moroccan lentil varieties (Bakria, Chakkouf, and Zaria). The values are the mean of three replicates ± standard deviation; *represents significant difference compared to the not inoculated control using ANOVA at P-value < 0.05.

Lentil variety	Isolates	Average number of the root nodules (SD)	Mean plant dry weight (g)	Average plant height (cm)	Average total N in leave (PPM)
Bakria	**Control**	0	0.01±0	5.33±0.58	6.27±0.3
	**318N211**	26.33* (±0.58)	*0.15±0.01	*26.33±2.31	*21.42±0.6
	**686N5**	*4.33±0.58	0.05±0.02	14.33±4.51	*18.23±0.2
	**115N2**	*14± 1	0.1±0.02	*19.33±3.06	6.82±4
	**1574N4**	*15±1	0.12±0.07	*17.67±11.06	*20.61±0.6
	**318N2111**	*32.33±2.52	*0.25±0.02	*32±3	*20.68±0.3
	**996N2**	*21±1	*0.35±0.03	*31.33±2.31	6.33±0.1
	**322N32**	*36±1	*0.52±0.03	*34±2	*20.74±0.3
	**1145N5**	*32±1	*0.39±0.04	*32.67±3.06	*21.05±0.6
	**1159N32**	*30.33±1.53	*0.37±0.08	*34.33±7.37	*21.07±0.1
	**938N3**	*27±1	*0.17±0.01	*28±1.73	*15.65±0.2
	**1159N24**	*24.33±1.15	*0.28±0.06	*31.67±7.23	7.22±2
	**996N5**	*31±1	*0.24±0.01	*31±1	*20.51±0.1
	**1159N52**	*4±1	0.11±0.03	19±4.36	*21.34±0.4
	**1145N1**	*16±1	*0.14±0.01	*24.33±2.08	*20.79±0.2
Chakkouf	**Control**	0	0.02±0.02	5.67±2.52	5.5±0.5
	**318N211**	*37±1	*0.3±0.05	26.33±3.06	*6.43±2.3
	**686N5**	*22±1	*0.32±0.09	*29±7.94	*16.98±0.2
	**115N2**	*14.33±0.58	*0.23±0.06	*28.33±7.09	*20.55±1
	**1574N4**	*22±1	0.19±0.06	25.33±8.14	*20.85±0.2
	**318N2111**	*30±1	*0.24±0.01	*31.67±1.53	13.55±0.6
	**996N2**	*22.67±0.58	0.13±0.05	22±8.89	*20.74±0.3
	**322N32**	*9±1	0.04±0.01	14.67±4.04	*19.66±1.5
	**1145N5**	*15.67±0.58	0.18±0.11	24±6.56	*18.32±0.4
	**1159N32**	*29.33±1.15	0.15±0.02	*28±3.61	*20.87±0.3
	**938N3**	*21±1	0.15±0.04	19.67±5.03	*21.27±0.5
	**1159N24**	*29±1	0.15±0.03	*25.67±5.51	*12.64±0.3
	**996N5**	*18±2	0.08±0.02	17.33±1.15	*20.48±0.1
	**1159N52**	*7±1	*0.21±0.1	21±10.15	*13.27±2

	**1145N1**	*15.33±0.58	*0.3±0.11	* 33.3±10.58	*20.57±0.1
	**Control**	0	0.06±0.04	5.8 ±1.04	5.35±0.7
	**318N211**	*13.33±0.58	0.21±0.07	*27.7 ±2.52	*16.42±0.9
	**686N5**	*5.33±1.53	0.03±0.02	8.33±1.53	7.42±1
	**115N2**	*8±1	0.08±0.07	13±10.58	*10.59±1.4
	**1574N4**	*12.67±0.58	0.1±0.03	21.33±6.35	*14.68±1.2
	**318N2111**	*15.33±0.58	*0.39±0.09	*27.33±6.35	*20.6±0.8
	**996N2**	*29.33±0.58	*0.26±0.01	*31.33±1.15	*20.48±0.6
**Zaria**	**322N32**	*16.67±0.58	0.09±0.03	21.67±8.74	7.29±0.8
	**1145N5**	*15.67±1.53	0.18±0.11	*28.67±0.58	*20.68±0.6
	**1159N32**	*26.67±1.53	0.13±0.01	*28±1	*21.82±1
	**938N3**	*23.33±1.53	0.15±0.03	25±5.29	7.54±0.9
	**1159N24**	*34.67±1.53	0.14±0.02	*26.67±2.89	6.29±0.5
	**996N5**	*33.33±1.53	*0.24±0.05	*27±6.08	*21.81±1.1
	**1159N52**	*13.67±1.53	0.15±0.09	21.33±12.5	*18.85±1.7
	**1145N1**	*13.67±0.58	0.12±0.08	22±13.53	*21.33±0.8
